# Non-causal relationship of polycystic ovarian syndrome with homocysteine and B vitamins: evidence from a two-sample Mendelian randomization

**DOI:** 10.3389/fendo.2024.1393847

**Published:** 2024-05-21

**Authors:** Nianjun Su, Jinsheng Li, Yubing Xia, Cuiyu Huang, Lei Chen

**Affiliations:** ^1^Department of Reproductive Health and Infertility, Guangdong Province Women and Children Hospital, Guangzhou, China; ^2^South China University of Technology School of Medicine, Guangzhou, China; ^3^Wurang Town Health Center, Zhaoqing, China; ^4^Department of Obstetrics and Gynecology, The Sixth Medical Center of People's Liberation Army (PLA) General Hospital, Beijing, China

**Keywords:** polycystic ovary syndrome, homocysteine, B vitamins, Mendelian randomization, instrumental variables

## Abstract

**Objective:**

Previous observational studies have identified a correlation between elevated plasma homocysteine (Hcy) levels and polycystic ovary syndrome (PCOS). This study aimed to determine whether a causal relationship exists between Hcy and PCOS at the genetic level.

**Methods:**

A two-sample Mendelian Randomization (TSMR) study was implemented to assess the genetic impact of plasma levels of Hcy, folate, vitamin B12, and vitamin B6 on PCOS in individuals of European ancestry. Independent single nucleotide polymorphisms (SNPs) associated with Hcy (n=12), folate (n=2), vitamin B12 (n=10), and vitamin B6 (n=1) at genome-wide significance levels (*P*<5×10^-8^) were selected as instrumental variables (IVs). Data concerning PCOS were obtained from the Apollo database. The primary method of causal estimation was inverse variance weighting (IVW), complemented by sensitivity analyses to validate the results.

**Results:**

The study found no genetic evidence to suggest a causal association between plasma levels of Hcy, folate, vitamin B12, vitamin B6, and PCOS. The effect sizes, determined through random-effect IVW, were as follows: Hcy per standard deviation increase, OR = 1.117, 95%CI: (0.842, 1.483), *P* = 0.442; folate per standard deviation increase, OR = 1.008, CI: (0.546, 1.860), *P* = 0.981; vitamin B12 per standard deviation increase, OR = 0.978, CI: (0.808, 1.185), *P* = 0.823; and vitamin B6 per standard deviation increase, OR = 0.967, CI: (0.925, 1.012), *P* = 0.145. The fixed-effect IVW results for each nutrient exposure and PCOS were consistent with the random-effect IVW findings, with additional sensitivity analyses reinforcing these outcomes.

**Conclusion:**

Our findings indicate no causal link between Hcy, folate, vitamin B12, vitamin B6 levels, and PCOS.

## 1 Introduction

Polycystic ovary syndrome (PCOS) is a complex endocrine metabolic disorder characterized by hyperandrogenemia, oligo- or anovulation, and polycystic ovarian morphology. According to the Rotterdam criteria, approximately 8%-13% of women globally are diagnosed with PCOS (1, 2). Beyond infertility, individuals with PCOS frequently experience long-term health issues, including obesity, insulin resistance, cardiovascular diseases, and other metabolic dysfunctions (3, 4). The precise origins of PCOS remain elusive; however, pathophysiological research indicates that it is a heterogeneous condition influenced by genetic predispositions, environmental factors, and hereditary components (4, 5). Recent studies have highlighted a notably increased risk of cardiovascular conditions such as coronary heart disease and stroke among those with PCOS (6–8) and have observed alterations in vascular endothelia associated with the syndrome (9). Two extensive cohort studies in Denmark have established that the risk of cardiovascular disease in PCOS is elevated, independent of body mass index (BMI) (10, 11). Some researchers have proposed viewing the cardiovascular risks associated with PCOS and its sequelae as a “risk enhancer” (12). Nevertheless, findings from recent Mendelian randomization (MR) studies challenge earlier clinical observations (13) by refuting a direct causal link between PCOS and major cardiovascular events like coronary heart disease and stroke (14), indicating that comorbid conditions of PCOS may significantly contribute to its long-term adverse effects.

Homocysteine (Hcy), a sulfur-containing amino acid, is an intermediate product in the metabolic conversion of methionine. Hyperhomocysteinemia (HHcy, defined as a plasma Hcy level ≥15 mmol/L) can result from low dietary intake of folate or vitamin B12, or from mutations in the MTHFR and CBS genes (15). A recent meta-analysis illustrated that the pooled prevalence of HHcy among Chinese females is 28%, indicating an upward trend (16). Furthermore, studies have uncovered a causal link between reduced vitamin B12 levels and an increased risk of PCOS (17), as vitamin B12 deficiency contributes to elevated Hcy levels. Various investigations have confirmed the association between higher Hcy levels and PCOS, demonstrating significant increases in both circulating plasma and follicular fluid Hcy levels in PCOS patients (18, 19). Research involving PCOS patients who experienced recurrent pregnancy loss (RPL) has shown that high levels of Hcy in serum and follicular fluid induce apoptosis in granulosa cells and impair villous angiogenesis, which may lead to defects in embryo implantation and early miscarriage (20). However, folate supplementation has been shown to mitigate the effects of Hcy (21). Elevated Hcy levels in PCOS patients have also been linked to poor oocyte maturation, reduced fertilization rates, and decreased embryo quality, thereby adversely affecting fertility (22, 23). Moreover, high Hcy levels show linkage with obesity, insulin resistance, and elevated androgen levels (24), contrasting with previous meta-analysis results (25). High Hcy levels are also related to insulin resistance and exacerbate hyperandrogenism, a key feature of PCOS (25). After adjusting for age, BMI, insulin resistance, and other variables, multivariable logistic regression analysis revealed that serum Hcy significantly increases the risk of PCOS [OR=1.172, CI: (1.032, 1.330)] (26). Consequently, Saadeh N et al. have noted that serum Hcy strongly correlates with PCOS and serves as an effective predictor for diagnosing PCOS [AUC=0.855, CI: (0.811, 0.898)] (27).

The relationship between elevated Hcy levels and the incidence of cardiovascular disease (CVD) has been substantiated by previous research (28). Additionally, the interplay between HHcy and biochemical HHcy may exacerbate cardiovascular risks in women with PCOS (29). Insulin resistance and HHcy are prominent features of PCOS, with insulin resistance prompting compensatory HHcy. It has also been suggested that endogenous opiates may contribute to HHcy in PCOS patients (30). Despite adjustments for age, BMI, insulin resistance, and other factors, serum Hcy levels remain significantly higher in PCOS patients, potentially increasing the risk of developing PCOS (26). While elevated Hcy levels are implicated in linking PCOS to cardiovascular incidents, the direct association between PCOS and cardiovascular outcomes continues to be a subject of debate, largely due to varying diagnostic approaches for PCOS and definitions of CVD (31). Thus, further research is imperative to elucidate the relationship between elevated Hcy levels and PCOS.

MR studies, through the natural grouping of instrumental variables (IVs), offer a robust method for addressing potential confounders and biases inherent in observational studies, establishing a reliable causal relationship between Hcy and PCOS at the genetic level, and also testing for reverse causality. This study aimed to determine whether there is a causal relationship between Hcy, folate, vitamin B12, and vitamin B6 and PCOS using MR analysis. This investigation is crucial for identifying potential causes of elevated Hcy levels in women with PCOS and the role of folate supplementation in managing elevated serum Hcy levels in this population.

## 2 Materials and methods

### 2.1 Data sources

**Figure 1:**
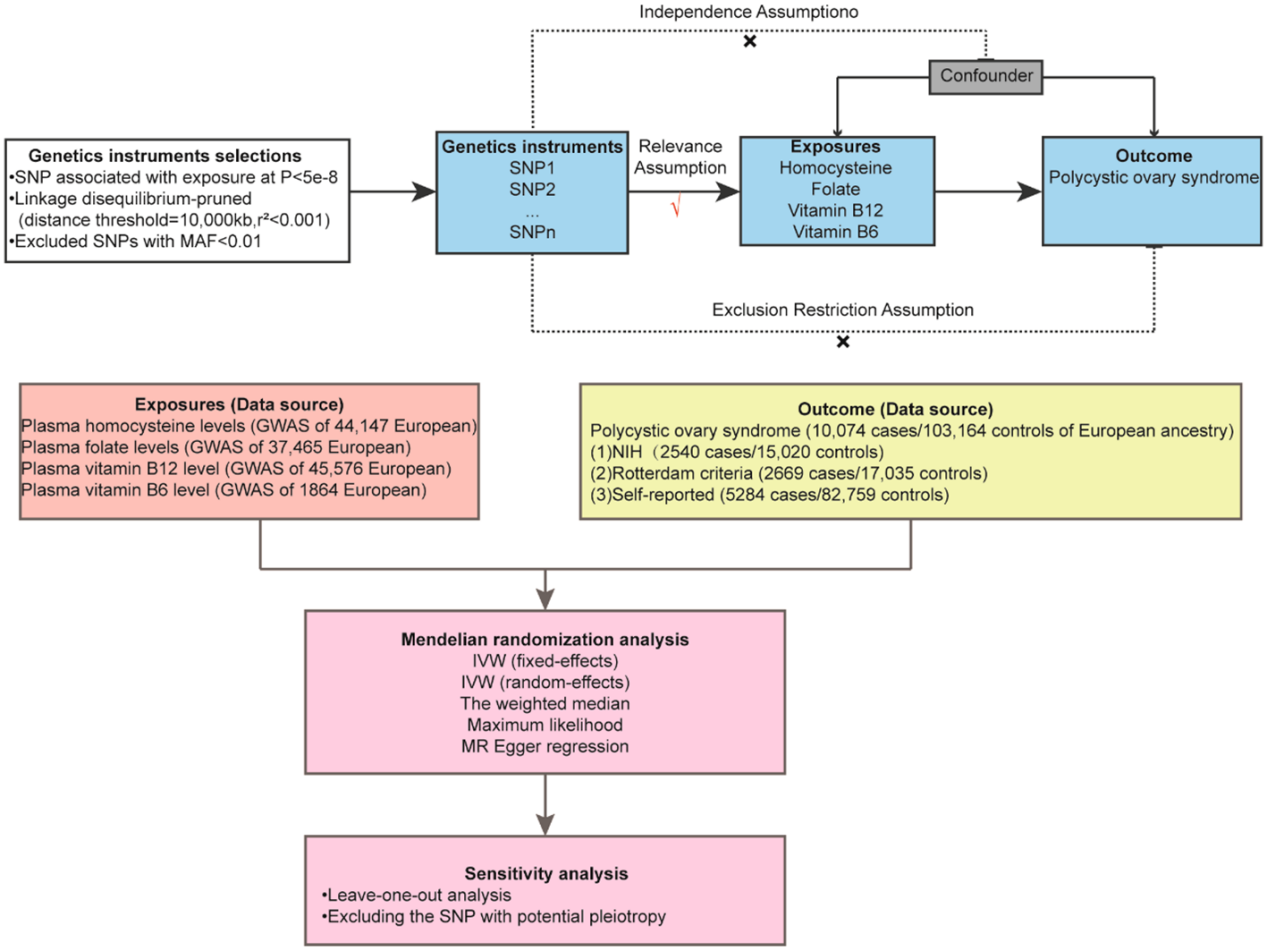
Overview Mendelian randomization analysis of plasma homocysteine, folate, vitamin B12, and vitamin B6 level with polycystic ovary syndrome.

Genome-wide association study (GWAS) data sources for Hcy, vitamin B6, vitamin B12, folate, and PCOS are readily accessible online (**Figure 1**). Single nucleotide polymorphisms (SNPs) associated with these variables were employed as IVs in this study. Ethical approval is not required for this study since it utilizes data collected from published studies and public databases. Detailed information regarding the ethical approval and informed consent for each subject can be found in the original publications where these data were first reported.

#### 2.1.1 Exposed data sources

SNPs associated with serum Hcy concentrations were selected from the largest genome-wide association meta-analysis conducted to date (44,147 individuals of European ancestry) (32). SNPs related to folate and vitamin B12 were derived from previous GWAS studies, incorporating 37,465 and 45,576 individuals of European descent, respectively (33). SNPs associated with vitamin B6 were obtained from earlier GWAS research involving 1,864 individuals of European descent (34, 35).

#### 2.1.2 Data source of outcome

SNPs associated with PCOS were extracted from the current largest GWAS, which included 10,074 cases and 103,164 controls from seven European cohorts involved in the 1000 Genomes Project or HapMap2 (36). The diagnostic criteria for PCOS were defined as follows: NIH criteria (2,540 cases/15,020 controls), Rotterdam criteria (2,669 cases/17,035 controls), and self-reported cases (5,284 cases/82,759 controls). SNPs related to insulin resistance and total testosterone levels were sourced from the IEU Open GWAS project. Additionally, SNPs related to obesity were obtained from FinnGen release 8 (http://r8.finngen.fi). The FinnGen project is a pioneering research initiative that combines genetic data with digital healthcare records from over 500,000 participants in Finnish biobanks (35).

### 2.2 Screening of IVs

Utilizing the 1000 European Genomic Reference Panels, we applied the PLINK clustering method to evaluate the linkage disequilibrium of SNPs and selected independent SNPs with no linkage disequilibrium as IVs. The selection criteria for these SNPs were as follows: [1] They must satisfy the independence hypothesis, with an r^2^<0.001, window size=10000kb and *P* value<5E-0^8^; [2] A minor allele frequency (MAF) of ≥0.01; [3] The absence of correlation with potential confounding factors, as verified through the PhenoScanner database (http://www.phenoscanner.medschl.cam.ac.uk) (37); [4] In cases where specific PCOS GWAS data for an SNP are unavailable, no proxy SNP is sought; [5] SNP harmonization was carried out to correct allelic orientation, and palindromic SNPs were excluded; [6] The strength of selected IVs was calculated utilizing the F-statistic and R^2^, where R^2^ = 2 × EAF × (1 - EAF) × β^2^/(2 × EAF × (1 - (EAF) × β^2 +^ 2 × EAF × (1 - EAF) × N × SE x β^2^] (EAF: effect allele frequency, β: beta, N: sample size, SE, standard error). The F-statistic was calculated as = (N-2) × R^2^/(1-R^2^) (N: sample size) (38). An F-statistic below 10 was considered indicative of a weak IV (39). The final selected SNPs are presented in **Supplementary Table S3**.

### 2.3 MR analysis

To investigate the causal relationship between Hcy levels and PCOS, we conducted a two-sample MR (TSMR) analysis. The foundational assumptions of MR include [1] a strong correlation between the IVs and the exposure, [2] no correlation between IVs and potential confounding variables, and [3] IVs influence the outcome exclusively through the exposure. The inverse variance weighting method (IVW) served as the primary analytical approach for MR analysis (40). Cochrane’s Q value was employed to evaluate the heterogeneity of SNP estimates. In the absence of significant heterogeneity (*P* < 0.05), a fixed-effect model was utilized. If heterogeneity was detected, a random-effects model was adopted (41). Several important sensitivity analyses were then implemented. The weighted median method provided an estimate consistent with IVW when the effective IV proportion exceeded 50% (42). MR-Egger, which uses the P-value of its intercept to assess horizontal pleiotropy, often yields a broad confidence interval due to its limited statistical efficiency (43). Furthermore, MR-PRESSO, grounded in the InSIDE hypothesis, was used to detect and correct for bias potentially introduced by pleiotropic outliers through a global test and outlier removal (44). The robustness of the primary results was confirmed through leave-one-out analysis, systematically excluding one SNP at a time. To mitigate potential confounding effects related to PCOS, SNPs were screened for confounding factors employing the Phenoscanner V2 database. Power analyses were performed using the mRnd network calculation tool (https://shiny.cnsgenomics.com/mRnd/). Additional MR analyses were implemented to further substantiate the relationship between Hcy, B vitamins, and PCOS symptoms. All statistical analyses were executed utilizing R software (version 4.3.1) with the “TwoSampleMR” and “MR-PRESSO” packages. A *P*-value of <0.05 was deemed statistically significant.

## 3 Results

### 3.1 Causal effects of Hcy on PCOS

**Figure 2:**
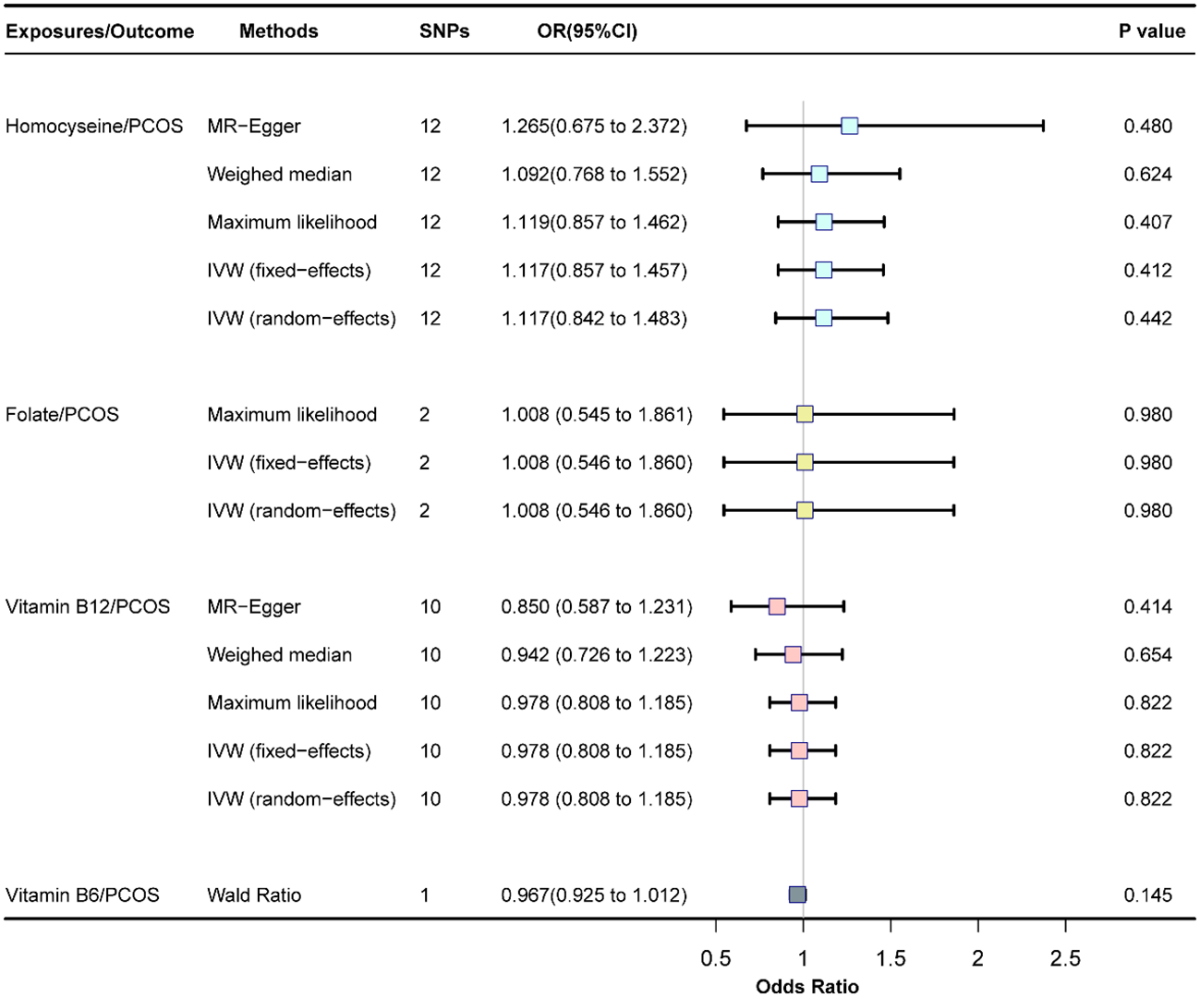
Association of homocysteine, folate, vitamin B12, and vitamin B6 with PCOS, SNP, single nucleotide polymorphism; OR, odds ratio; Cl, confience interval; PCOS, polycystic ovary syndrome.

**Figure 3:**
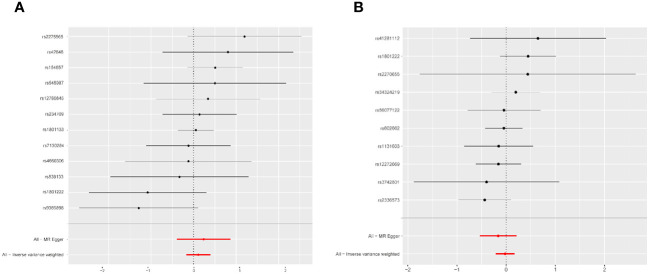
Forest plot of the potential effects of homocysteine associated SNPs and vitamin B12-associated SNPs on PCOS. **(A)** Homocysteine-associated SNPs on PCOS. **(B)** Vitamin B12 associated SNPs on PCOS. PCOS, polycystic ovary syndrome; MR, mendelian randomization; All-Inverse variance weighted, random effects inverse variance weighted analysis.

Fixed-effects models analyzed through IVW revealed that genetically predicted Hcy levels were not significantly associated with PCOS [odds ratio (OR) = 1.117, CI: (0.857, 1.457), *P* = 0.413]. These results were consistent with those obtained from the random-effects model IVW analysis [OR = 1.117, CI (0.842, 1.483), *P* = 0.442]. Further analyses using MR-Egger, the weighted median method, and maximum likelihood estimation supported these findings (**Figure 2**). The potential causal effects of SNPs associated with Hcy on PCOS were further explored and are depicted in **Figure 3**. A funnel plot analysis indicated that the effects of Hcy were symmetrically distributed, suggesting the absence of directional pleiotropy (**Supplementary Figure S1**). Additionally, Cochran’s Q test for heterogeneity was not significant (Q = 12.501, *P* = 0.327), indicating a lack of variance across studies. No evidence of horizontal pleiotropy was detected (*P* for intercept = 0.670), and MR-PRESSO analysis confirmed the absence of outliers (Global test *P*-value = 0.406) (**Supplementary Table S5**).

### 3.2 Causal effect of folate on PCOS

The fixed-effects model of IVW analysis revealed that genetically predicted folate levels were not significantly associated with PCOS [OR = 1.008, CI: (0.546, 1.860), P = 0.981]. This result was corroborated by the random-effects model IVW analysis, with consistent findings reported across all methods (**Figure 2**).

### 3.3 Causal effects of vitamin B12 on PCOS

The fixed-effects model of IVW analysis indicated that genetically predicted vitamin B12 levels were not associated with PCOS [OR = 0.978, CI:(0.808,1.185), *P* = 0.823]. These findings were consistent with the random-effects model IVW analysis. Further analyses using MR-Egger, the weighted median, and maximum likelihood confirmed these results, as shown in **Figure 2**. The potential causal effect of SNPs associated with vitamin B12 on PCOS was assessed and detailed in **Figure 3**. The funnel plot analysis demonstrated symmetrically distributed effects of vitamin B12, with no evidence of directional pleiotropy (**Supplementary Figure S1**). Moreover, Cochran’s Q test revealed no significant heterogeneity (Q = 7.470, *P* = 0.588), and there was no detection of horizontal pleiotropy (*P* for intercept = 0.410) or outliers in the MR-PRESSO analysis (global test *P* = 0.583) (**Supplementary Table S5**).

### 3.4 Causal effects of vitamin B6 on PCOS

The Wald Ratio analysis for genetically predicted vitamin B6 levels found no significant correlation with PCOS [OR=0.967, CI:(0.925,1.012), *P* = 0.145], as displayed in **Figure 2**. Due to the use of only one SNP in the IV set for vitamin B6, further pleiotropic tests and sensitivity analyses were not feasible.

### 3.5 Sensitivity analysis

**Figure 4:**
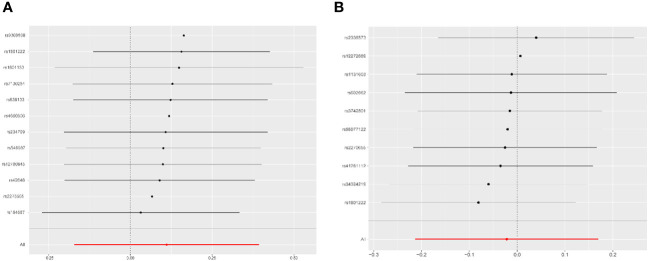
Leave-one-out analysis for the association of homocysteine and vitamin B12 with PCOS. **(A)** Homocysteine оn PCOS. **(B)** Vitamin B12 оn PCOS. PCOS, polycystic ovary syndromme.

Leave-one-out analysis demonstrated that the effects of Hcy and vitamin B12 on PCOS remained unchanged with the sequential exclusion of any single SNP (**Figure 4**). The power assessment results are detailed in **Supplementary Table S4**. Furthermore, a thorough review of each SNP’s pleiotropy using the Phenoscanner V2 database revealed a previously identified Hcy SNP (rs548987) associated with BMI, which showed a significant association with PCOS (**Supplementary Table S2**). Reevaluation of the effect sizes, after excluding this SNP, yielded consistent results (**Supplementary Figure S2**). Additionally, we identified a causal association between vitamin B12 and obesity, evidenced by an OR of 0.938 (95% CI: 0.891–0.988, *P* = 0.013). No causal relationships were found between Hcy, B vitamins, and other metabolic traits such as insulin resistance, obesity, or total testosterone levels (**Supplementary Figure S3**). A reporting checklist for this TSMR study is provided in **Supplementary Table S1**.

## 4 Discussion

In this study, we investigated the potential causal associations between plasma levels of Hcy, folate, vitamin B12, and vitamin B6 and the risk of PCOS. Our findings indicated that there was no substantial evidence to suggest that genetically predicted levels of Hcy, folate, vitamin B12, and vitamin B6 were causal factors for PCOS.

Our investigation found no genetic causal connection between elevated genetically predicted plasma Hcy levels and the incidence of PCOS. This conclusion contrasts with several observational studies that have reported increased plasma Hcy levels in women with PCOS (18, 27, 45). Homocysteine thiolactone, an active metabolite of Hcy, has been demonstrated to disrupt tyrosine phosphorylation in the insulin receptor β-subunit and related substrates, hindering phosphatidylinositol 3-kinase activity and subsequently reducing insulin-mediated glycogen synthesis, a key factor in the development of insulin resistance (46). Experimental studies in a PCOS mouse model indicate that HHcy could intensify insulin resistance and inflammation in adipose tissue by altering macrophage M2 polarization via estrogen inhibition (19). Common metabolic disturbances associated with PCOS, such as insulin resistance and hyperinsulinemia, have also been associated with elevated Hcy levels, which are further linked to heightened risks of hyperinsulinemia and atherosclerosis (47). Biochemical hyperandrogenism, another hallmark of PCOS, significantly correlates with increased HHcy risks, showing an effect size of 2.24 (95% CI: 1.26–4.01) (29). Ting Li et al. found that androgens may escalate Hcy levels by inhibiting the mammalian target of rapamycin pathway in granulosa cells from PCOS-affected mice (48). It is hypothesized that the higher incidence of HHcy observed in the PCOS population might reflect an increased mutation rate of the MTHFR gene, particularly in Asian populations, where polymorphisms such as MTHFR rs1801131 and MTHFR rs1801133 could be contributing factors to elevated Hcy levels in PCOS (49). Despite these associations, our sensitivity analysis found no evidence of a causal link between Hcy levels and the risk of PCOS or its related symptoms, including insulin resistance, obesity, and total testosterone levels.

The relationship between vitamin B12 supplementation and the risk of PCOS remains a topic of debate. Vitamin B12 acts as a methyl donor in conjunction with folate in the methylation process. A deficiency in vitamin B12 can impede the Hcy remethylation pathway, leading to increased levels of circulating Hcy (50). Previous randomized controlled trials (RCTs) have demonstrated that supplementation with vitamin B12 and folate effectively reduces blood Hcy levels in PCOS patients (51). However, our results indicated no significant causal relationship between genetically predicted vitamin B12 levels and the risk of PCOS. Contrastingly, another recent MR study (17) reported findings that suggest genetically predicted vitamin B12 may reduce the risk of PCOS (IVW-MR: OR = 0.753, CI = [0.5688–0.998], *P* = 0.048) and obesity (IVW-MR: OR = 0.917, CI = [0.843–0.995], *P* = 0.037). These discrepancies between studies could be attributed to several factors: First, Shen JY et al. did not apply Bonferroni or False Discovery Rate (FDR) corrections for P values, which could increase the risk of false positives due to multiple testing. Second, the inconsistency in GWAS data sources for PCOS may also influence outcomes. Shen JY et al. utilized data from the FinnGen database, which included 642 cases and 118,228 controls, whereas our study used data from the largest PCOS GWAS to date, Apollo, with 10,074 cases and 103,164 controls. Despite these differences, our sensitivity analysis revealed that vitamin B12 deficiency was associated with an increased risk of obesity, aligning with previous findings (17).

Previous research has established that a deficiency of vitamin B6 in the general population leads to an elevated Hcy levels (52). Vitamin B6 is crucial for the catabolic pathway of Hcy, catalyzing the conversion to cysteine. However, in this study, only one effective IV for vitamin B6 was identified, which may limit the statistical power of our findings and affect the reliability of the observed negative causal relationship between vitamin B6 and PCOS (42).

folate supplementation is a recognized therapeutic strategy to reduce elevated HHcy levels. As a vital component in Hcy metabolism, a decrease in serum folate is one of the primary causes of HHcy. Recent meta-analyses and RCTs have shown that folate supplementation can improve insulin resistance and glucose metabolism in individuals (53). Specifically, two prior RCTs demonstrated that daily supplementation of 5 mg of folate for eight weeks significantly reduced Hcy levels, inflammatory markers, and HOMA-IR scores in PCOS patients (54, 55). The underlying mechanism is likely related to folate’s role in enhancing the DNA methylation of genes involved in metabolic regulation (56), which helps to mitigate cellular and protein damage caused by oxidative stress and maintains endothelial function through single-carbon metabolism (57). Moreover, a recent systematic review involving eight RCTs highlighted that folate supplementation not only improves BMI in women with HHcy but also in women with PCOS (58); Folate supplementation could be particularly beneficial for obese PCOS patients with HHcy.

This study’s primary strength lies in its utilization of the largest available GWAS data on Hcy-SNPs and PCOS-SNPs, employing a MR design. This approach enhances the causal inference of the relationship between Hcy, B vitamins, and PCOS by reducing residual confounding and other biases. However, the study is not without limitations. Firstly, the number of genome-wide association studies and single nucleotide polymorphisms available for analysis in PCOS is relatively limited. Azziz R has indicated that the identified loci might account for less than 20% of the heritability of PCOS, which raises concerns about the comprehensiveness of the results obtained from MR analysis (59). Secondly, the current GWAS data for PCOS do not include subtype classification based on the Rotterdam recommendations, which restricts our ability to discern potential associations between Hcy exposure and specific PCOS subtypes. Thirdly, this study is based primarily on data from European populations, limiting its applicability to other ethnic groups. Such geographical and genetic specificity might hinder the generalization of the findings to broader, more diverse populations. Fourthly, while we have elucidated the relationship between Hcy and PCOS from a genetic standpoint, it is important to recognize that PCOS is a complex endocrine and metabolic disorder influenced by genetic, metabolic, and environmental factors. Consequently, our results, focused solely on genetic contributions, may present certain limitations in fully capturing the multifaceted nature of PCOS.

## 5 Conclusions

The findings from our MR analysis currently provide no evidence to support a causal relationship between genetically predicted Hcy levels and PCOS. Additionally, supplementation with folate and vitamin B12 does not appear to reduce the risk of PCOS. Given these results, further research is essential to explore the impact of Hcy on various subtypes of PCOS, as defined by the Rotterdam criteria. Such investigations are critical for enhancing our understanding of PCOS and could potentially lead to more effective treatment strategies for PCOS patients exhibiting clinical HHcy.

## Data availability statement

The original contributions presented in the study are included in the article/**Supplementary Material**. Further inquiries can be directed to the corresponding authors.

## Ethics statement

Ethical approval was not required for the study involving humans in accordance with the local legislation and institutional requirements. Written informed consent to participate in this study was not required from the participants or the participants’ legal guardians/next of kin in accordance with the national legislation and the institutional requirements.

## Author contributions

NS: Writing – review & editing, Writing – original draft, Supervision, Project administration, Conceptualization. JL: Writing – review & editing, Writing – original draft, Formal Analysis, Conceptualization. YX: Visualization, Writing – original draft. CH: Writing – review & editing. LC: Writing – review & editing, Visualization, Supervision, Project administration, Methodology, Formal analysis, Conceptualization.
